# Supplementary material for: Non-communicable diseases and inequalities increase risk of death among COVID-19 patients in Mexico
Source: PLoS One. 2020 Oct 8;15(10):e0240394. doi: 10.1371/journal.pone.0240394 (PMC7544063; doi:10.1371/journal.pone.0240394)
Supplement: Attachment — Submitted filename: Response to reviewers v28Jul2020.docx [file pone.0240394.s001.docx]

In addition to revisions made base on reviewers’ comments, we also update the data in the analysis to the July 7 release, increasing substantially the number of observations

| Comment | Response / Action |
| --- | --- |
| Journal requirements |  |
| Please ensure that your manuscript meets PLOS ONE's style requirements, including those for file naming. | OK, done |
| In your Methods section, please provide additional information about the methodology of your study. Please ensure you have provided sufficient details to replicate the analyses such as: a) the date on which the dataset was accessed, b) a description of all variables included in the analysis, and explanation on how these were categorised. Moreover, please refer to the specific statistical analyses performed as well as any post-hoc corrections to correct for multiple comparisons. If these were not performed please justify the reasons | We added the requested details to the manuscript. We did the Benjamini–Hochberg procedure for a false discovery rate approach. |
| We suggest you thoroughly copyedit your manuscript for language usage, spelling, and grammar. If you do not know anyone who can help you do this, you may wish to consider employing a professional scientific editing service. | Dr. Bertozzi copyedited the manuscript. |
| Reviewer 1 |  |
| In general, the manuscript is brief, and so I did have some questions on the methods. It would be important to have a clearer understanding of the data source. Who is included, who might be excluded? Are there any data verification steps? As the data are from an administrative source, it is important the authors share as much information as possible about the reliability of the information. I am not familiar with Mexican administrative datasets. | It is an administrative dataset that includes all tested individuals in the country. For the models, we are only including those that tested positive for SARS-CoV-2. We added additional detail on the data available and included in limitations that data were posted by the government as is. |
| I am struggling to understand the 'pool' of tested patients adequately. What were the country criteria for testing? Where were tests conducted? Were these limited to hospital settings? This may of course be very relevant to the subsequent indigenous language analyses. | We included additional detail on the countries approach for testing. All facilities are mandated to test severe cases of viral respiratory infection, so that includes those attending indigenous individuals. |
| The logistic regression models were simple - exploring risks of hospital admission, intubation or death. The models use outcomes in a binary manner - but my evaluation of the dataset led me to believe dates were included for death, so survival models would have been an option. Was this considered? | Survival analysis will be an option once more detail is included in the dataset. We thank the reviewer for the suggestion that we may implement in a following manuscript as more detailed data became available. |
| In the logistic regression, I would be keen to understand the detail of the models controlling for hospitalisation? Surely it would have been more appropriate to analyse intubation only amongst hospitalised patients? Maybe this was the case - but I was not sure. | Yes, you are right, controlling for hospitalization is for death as outcome, but intubation is analyzed only for those hospitalized. We clarify that in the manuscript: |
| Could the authors justify the choice of confounders (were these simply reflective of available data)? | We included as much information as available in the dataset. |
| The results tables need clearer labelling. I presumed age was mean +/- SD... but this should be explicitly stated. Similarly, variables like 'obesity' need defining clearly. | Thanks, we corrected the labeling. |
| The rate of admission in the non-sars-cov2 group struck me as high. Perhaps the authors could add the sensitivity of their testing kits in Mexico? | Testing in the country is performed for symptomatic individuals, so those that tested negative are likely to have another respiratory infection that would explain hospitalization among this group. |
| The second table should ideally present the odds ratios alongside 95% confidence intervals. There is an extraordinary amount of hypothesis testing being done, and I would be more comfortable with fewer p values presented! | We included the 95% CI in the table, and also implemented a false discovery rate following the Benjamini–Hochberg procedure in order to confirm significance. |
| Along the same lines, there appear to be some unusual interactions, possibly with effect modification, between asthma, COPD and smoking. My instinct these are confounded by age. I would like to know how the authors explored these associations. | We added in the discussion the hypothesis that these individuals sought care relatively earlier and that facilitated a better prognosis. |
| Ideally I would have liked to see the logistic regression data accompanied by absolute numbers, to be able to appreciate absolute v relative risks. | We added absolute numbers to table 2. |
| The discussion is brief (I appreciate this is a concise report). However, my sense is that the really novel aspects of the results are the descriptives of the cohort in Mexico in terms of burden of NCD, plus the work around deprivation and indigenous language. The observations around age and comorbidity and their relationship with SARS-CoV-2 outcome are well described now | Thanks for the comment, we reviewed the discussion as suggested. |
| There are some minor edits required for typographic errors, but overall the language and style are good. | Thanks, we copyedited the revised manuscript. |
| Reviewer 2 |  |
| I think that this letter could be greatly improved with better background and methods, mainly by considering previously existing articles and by clarifying and improving the methodological approach. It is noteworthy to mention that a similar (smaller) study was also published, without peer review process, (doi: 10.1101/2020.05.11.20098145), although it did not consider any socioeconomic indicator regarding poverty. | Thanks for the comment; we have edited the manuscript to improve background and methodology as suggested. |
| The abstract should be re-written. There are a lot of different messages and outcomes; The rationale and conclusions should be more aligned with the main outcomes. | We edited the abstract as suggested. |
| According to the main outcomes presented, it seems that the study aims to assess risk factors, including a socioeconomic indicator, for (1) hospitalization, (2) intubation and (3) death. Having said this, the goals should be redefined accordingly. Several already published studies with similar aims should also be referred in this section. Some examples (doi): 10.1136/bmj.m1985, 10.1080/13685538.2020.1774748, 10.1101/2020.05.06.20092999, 10.1002/jmv.26050 or 10.1016/j.jinf.2020.04.021. Other literature on inequalities and COVID-19 could also be considered. | Thanks for the comment; we reviewed the background section to incorporate this suggestion. |
| The sample considered in this study should be “patients with COVID-19 (confirmed cases with SARS-CoV-2)”. Why only consider adults as 20 years old or older? | We excluded under 20 individuals due to our interest on NCDs |
| There is no need to compare patients that tested positive and with those that tested negative for SARS-CoV-2 as it does not add information. Thus, the first part of the Results section can be deleted. Table 1 (descriptive analysis) should be reviewed considering the correct outcomes. | We believe it is informative in terms of comparing NCD prevalence among those groups. |
| Reference to “co-morbidities” or “comorbidities” should be uniformed throughout the manuscript. | Corrected |
| Information on data sources and quality should be clearly stated, as well as how missing data was handled (even if in supplementary material). | We included further detail on the dataset. Also, missing data were negligible, so for the regression analysis we excluded observations with missing values, that represented less than 0.5% of observations. |
| Regarding inequalities analysis, other methods could be considered. For example, why consider 50% on poverty as the cut-off instead of quintiles? | Thanks for the comment; in the revised version we are using quintiles of poverty share. |
| To estimate causation, other options might be considered or discussed in the Discussion (as limitations); e.g. directed acyclic graphs. Were all the analyzed variables included in the regression analyses? Any kind of univariate analysis firstly? For the logistic regression, 95% CI must be presented. | Thanks for the comment. We were not intending to estimate causation but association. We will explore in future analyses DAG. We added 95% CI in table 2. |
| Table 2 – It should be self-explanatory. 95% CI should be presented. The presentation could also be improved. Outcomes should be (1) hospitalization, (2) intubation and (3) death | Corrected as suggested. |
| Discussion should be more focused on the aforementioned aims (risk factors, including socioeconomic, for these outcomes) of this study, including comparison with the existing literature. | Thanks, we reviewed the discussion accordingly |
| Other limitations should be clearly presented (e.g. ecological fallacy of considering municipalities as socioeconomic indicators instead of individual information). | We clarify in the manuscript that we are using share of poverty at the municipality level, and also mention this under limitations. |
| Reviewer 3 |  |
| Reference 6 is neither complete nor in the journal format. Maybe it refers to the reference: Barquera S, Campos-Nonato I, Hernández-Barrera L, Pedroza A, Rivera-Dommarco JA. Prevalencia de obesidad en adultos mexicanos, 2000-2012 [Prevalence of obesity in Mexican adults 2000-2012]. Salud Publica Mex. 2013;55 Suppl 2:S151-60. Spanish. PMID: 24626691. | Corrected, thanks. |
| Some typographical errors are identified in reference 12. Correct please. | Corrected, thanks |
| Reviewer 4 |  |
| 1. Authors incorporate multilevel data regarding poverty status and comment that "In order to take into account local epidemic dynamics, estimations assumed that observations are clustered at the municipality level"; however, no specific description of the statistical technique utilized is being referred. Was this a mixed effects logistic model? Please specify. | Thanks for the comment. In the revised version we are using a mixed effects logistic model and described this in the methods sections. |
| 2. In the discussion section authors refer that severity of disease cannot be specified given available data but that is inaccurate. Previous definitions of severe COVID-19 have been based on composite outcomes of death, ICU admission and intubation, all of which are available for the Mexican Health Ministry Dataset (doi:10.1001/jamainternmed.2020.2033). Please comment on this and consider including an outcome related to severe COVID-19. | The comment on severity is related to the fact that criteria for hospitalization may be variable across facilities, as well as it is variable when individuals approach health services after symptoms. The suggested index included laboratory and image results that are not available in the dataset. |
| 3. A main concern of the present work is the definition of inequities. Whilst inarguably, poverty is a main driver of social inequity, there are additional factors which condition in equity and are better captured by other metrics measured by CONEVAL (http://sticerd.lse.ac.uk/dps/case/cp/CASEpaper205.pdf, https://www.coneval.org.mx/Medicion/IRS/Paginas/Que-es-el-indice-de-rezago-social.aspx). Authors are concouraged to either consider additional multidimensional metrics to be able to fully capture inequality or refer to their findings in relation to poverty. | The Mexican poverty measure it’s a multidimensional measure that captures even more dimensions than the suggested index. The index would be a better option if we could implement the analysis at the census tract level but unfortunately data from the Mexican government for COVID-19 cases are only identified up to municipality level.  It is also important to mention that we are referring to health inequities. |
| 4. Authors should comment on how the approach to case tracing and confirmation in Mexico might bias their results towards high-risk cases. Given that most cases which are being tested are symptomatic cases from key risk groups, interpretation of findings should be framed in this sense. | Thanks for the comment; we added a para on this as limitation: |
| 5. In the statistical analysis section it is asserted that "In addition to demographic characteristics and comorbidities, we also included state of residence to control for variations related to differences between states". This contradicts the previous comment that obervations were clustered to accunt for local dynamics. Simply adjusting for state of occurence controls some variability but the ideal model would incorportate this as a random effect within a mixed effects framework. These differences are relevant and a mixed effect model would effectively control these variabilities. | Thanks for the comment; we re-estimated the models using mixed effects model with municipality as a random effect. |
