# Supplementary material for: Non-communicable diseases and inequalities increase risk of death among COVID-19 patients in Mexico
Source: PLoS One. 2020 Oct 8;15(10):e0240394. doi: 10.1371/journal.pone.0240394 (PMC7544063; doi:10.1371/journal.pone.0240394)
Supplement: Attachment — Submitted filename: Reply to comments 21Sep.docx [file pone.0240394.s002.docx]

We thank the reviewer for the comments, that we addressed in this revised version. In the table below we included the answers to each comment.

In addition to revisions made base on reviewer’s comments, we also update the data in the analysis to the September 17 release, increasing substantially the number of observations

| Comment | Answer / action |
| --- | --- |
| I would reinforce that it would be useful to mention that a similar (without peer review process) study was also published (doi: 10.1101/2020.05.11.20098145), although with a small number of patients and not consider any socioeconomic indicator regarding poverty. | We included this reference now in the intro. |
| Please do not use inequality and inequity interchangeably | Corrected, thanks |
| Please do a proof-reading once again. | Thanks; we did an additional proof-reading. |
| “Other positively associated conditions that are also major contributors to the global burden of disease [7]” – it seems that it is not referring COVID-19 severity (?) | They are related to severity; we reformulated the wording for clarity |
| “Diabetes is the single largest contributor to disease burden in Mexico, with a prevalence of about 15% of the adult population. Hypertension is also very important, with a prevalence of 30%.” – please use references to support this | Thanks, we now included references for this. |
| “The triad of NCD, inequality and COVID doesn’t occur in the context of a high-functioning health care system, but rather one that is characterized by fragmentation, under-funding, lax regulatory oversight and a high proportion of poorly trained health personnel. The unfortunate result is low average health care quality which translates into unacceptably high mortality rates for severe COVID-19 disease.” – this triad exist in every countries with NCD, inequalities (depending on the cut-off) and with COVID-19, which correspond to almost every countries in the world. Please rephrase. | Agree; the intended meaning was to explain that this situation occurred in Mexico with an already challenged health system. We rephrased this. |
| Please reformulate the aim of the study as suggested before. | We reformulated to:  Using data from SARS-CoV-2 positive individuals in Mexico, our aim is to assess NCDs and socioeconomic status as risk factors for COVID-19 severe outcomes, in particular hospitalization, intubation and death |
| Missing data – It would be important to clearly state this with more detail. | Additional detail is now included in the revised version.  Overall, for the regression analysis, 3.5% of observations were excluded (23,037) due to missing values. The incidence of complications (hospitalization, intubation, death) among the excluded observations were similar to the included observations. |
| Have the quintiles such an exact distribution (i.e. 20%, 40%, …)? | No; as mentioned in the manuscript, are based on the % of the population living in poverty, i.e. all municipalities with up to 20% of the population living in poverty are part of the group 0% to 20%. |
| Table 2 – It seems still quite confusing, e.g. stating “probability” instead of “Odds”. | We changed to “odds” in the revised version |
